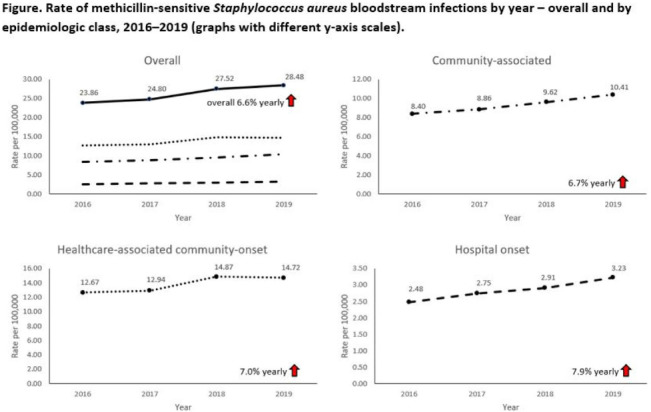# Increases in methicillin-sensitive Staphylococcus aureus bloodstream infection incidence, 2016–2019

**DOI:** 10.1017/ash.2022.179

**Published:** 2022-05-16

**Authors:** Kelly Jackson, Joelle Nadle, Susan Ray, Ruth Lynfield, Ghinwa Dumyati, Marissa Tracy, William Schaffner, David Ham, Isaac See

## Abstract

**Background:** Incidence of methicillin-sensitive *Staphylococcus aureus* (MSSA) bloodstream infections (BSIs) in the United States during 2012–2017 has been reported to have been stable for hospital-onset BSIs and to have increased 3.9% per year for community-onset BSIs. We sought to determine whether these trends continued in more recent years and whether there were further differences within subgroups of community-onset BSIs. **Methods:** We analyzed CDC Emerging Infections Program active, population- and laboratory-based surveillance data during 2016–2019 for MSSA BSIs from 8 counties in 5 states. BSI cases were defined as isolation of MSSA from blood in a surveillance area resident. Cases were considered hospital onset (HO) if culture was obtained >3 days after hospital admission and healthcare-associated community-onset (HACO) if culture was obtained on or after day 3 of hospitalization and was associated with dialysis, hospitalization, surgery, or long-term care facility residence within 1 year prior or if a central venous catheter was present ≤2 days prior. Cases were otherwise considered community-associated (CA). Annual rates per 100,000 census population were calculated for each epidemiologic classification; rates of HACO cases among chronic dialysis patients per 100,000 dialysis patients were calculated using US Renal Data System data. Annual increases were modeled using negative binomial or Poisson regression and accounting for changes in the overall population age group, and sex. Descriptive analyses were performed. **Results:** Overall, 8,344 MSSA BSI cases were reported. From 2016–2019 total MSSA BSI rates increased from 23.9 per 100,000 to 28.5 per 100,000 (6.6% per year; *P* < .01). MSSA BSI rates also increased significantly among all epidemiologic classes. HO cases increased from 2.5 per 100,000 to 3.2 per 100,000 (7.9% per year; *P* = .01). HACO cases increased from 12.7 per 100,000 to 14.7 per 100,000 (7.0% per year; *P* = .01). CA cases increased from 8.4 per 100,000 to 10.4 per 100,000 (6.7% per year; *P* < .01) (Fig. 1). Significant increases in MSSA BSI rates were also observed for nondialysis HACO cases (9.3 per 100,000 to 11.1 per 100,000; 7.8% per year; *P* < .01) but not dialysis HACO cases (1,823.2 per 100,000 to 1,857.4 per 100,000; 1.4% per year; *P* = .59). Healthcare risk factors for HACO cases were hospitalization in the previous year (82%), surgery (31%), dialysis (27%), and long-term care facility residence (19%). **Conclusions:** MSSA BSI rates increased from 2016–2019 overall, among all epidemiologic classes, and among nondialysis HACO cases. Efforts to prevent MSSA BSIs among individuals with healthcare risk factors, particularly those related to hospitalization, might have an impact on MSSA BSI rates.

**Funding:** None

**Disclosures:** None

**Figure:**